# Multimodal Management of Colorectal Liver Metastases: State of the Art

**DOI:** 10.3389/or.2023.11799

**Published:** 2024-01-04

**Authors:** Elisabetta Filoni, Vittoria Musci, Alessia Di Rito, Riccardo Inchingolo, Riccardo Memeo, Francesco Mannavola

**Affiliations:** ^1^ Interdisciplinary Department of Medicine, University of Bari “Aldo Moro”, Bari, Italy; ^2^ Division of Medical Oncology, A.O.U. Consorziale Policlinico di Bari, Bari, Italy; ^3^ Radiotherapy Unit, P.O. “Mons A.R. Dimiccoli”, Barletta, Italy; ^4^ Unit of Interventional Radiology, “F. Miulli” General Regional Hospital, Acquaviva delle Fonti, Italy; ^5^ Unit of Hepato-Pancreatic-Biliary Surgery, “F. Miulli” General Regional Hospital, Acquaviva delle Fonti, Italy

**Keywords:** liver metastases, colorectal cancer, CRC, chemotherapy, multimodal management

## Abstract

Liver is the most common site of colorectal cancer (CRC) metastases. Treatment of CRC liver metastases (CRLM) includes different strategies, prevalently based on the clinical and oncological intent. Valid approaches in liver-limited or liver-prevalent disease include surgery, percutaneous ablative procedures (radiofrequency ablation, microwave ablation), intra-arterial perfusional techniques (chemo-embolization, radio-embolization) as well as stereotactic radiotherapy. Systemic treatments, including chemotherapy, immunotherapy and other biological agents, are the only options for patients with no chance of locoregional approaches. The use of chemotherapy in other settings, such as neoadjuvant, adjuvant or conversion therapy of CRLM, is commonly accepted in the clinical practice, although data from several clinical trials have been mostly inconclusive. The optimal integration of all these strategies, when applicable and clinically indicated, should be ever considered in patients affected by CRLM based on clinical evidence and multidisciplinary experience. Here we revised in detail all the possible therapeutic approaches of CRLM focusing on the current evidences, the studies still in progress and the often contradictory data.

## Introduction

Colorectal cancer (CRC) is one of the most common malignancies [1] accounting 10% of global cancer incidence and 9.4% of cancer deaths worldwide [2]. More than half of patients develops metastases from CRC and the majority of them carry liver metastases (CRLM) [3, 4].

Fifteen to 25% of patients with CRC have synchronous (S)-CRLM, while another 20% will develop metachronous (M)-CRLM within 3 years from diagnosis [5]. Synchronous CRLM refers to those detected simultaneously with the primary tumor or within 6 months from diagnosis, while M-CRLM appear more than 6 months after primary tumor. Prognosis of patients with S-CRLM is generally worse as compared to M-CRLM [6–8], although several other factors, including the presence of node metastasis, number of CRLM (>5) and carcinoembryonic antigen (CEA) levels above 70 ng/mL, are prognostically relevant [9].

Different therapeutic options for CRLM are available, although radical resection remains preferable as it permits better results in terms of survival [4]. In this context, several clinical scores are available to stratify both prognosis and risk of recurrence after CRLM resection. Some of these are quite old since they are based on data from patients undergoing surgery before 2000 and are considered obsolete for current clinical practice. These scores (Table 1), including Fong [10], Nordlinger [11], Nagashima [12] and Konopke [13], evaluate different risk factors, including Disease Free-Interval (DFI), number and size of LM, presence of node metastasis, tumor staging, age and pre-operative CEA/Ca19.9 levels. Primary tumor location (sidedness) was also identified as relevant prognostic factor in CRLM; patients with CRLM from right-sided colon cancer, indeed, experience worse survivals after hepatic resections, as compared to left-side patients [15]. More recently, new risk scores have been validated, such as the CERR score [14] exploring different molecular factors, including mutations of RAS/BRAF, tumor protein P53 (TP53) and SMAD Family Member 4 (SMAD4) genes that were all associated with a poor prognosis [16–18]. The presence of high microsatellite instability (MSI-H) is also considered a negative prognostic factor in terms of tumor behavior, despite it is a strong positive predictive factor for response to immunotherapy with checkpoint inhibitors ensuring impressive responses and long survivals [19, 20].

**TABLE 1:** Clinical risk score of recurrence after CRLMs resection.

Risk factors	Fong [10]	Nordlinger [11]	Nagashima [12]	Konopke [13]	CERR [14]
DFI	<12 months	<24 months	—	Synchronous LMs	—
Number of LMs	>1	>3	>1	>3	>1
LM size	>5 cm	>5 cm	>5 cm	—	—
N stage	≥N1	≥N1	≥N1	—	≥N1
T stage	—	pT4	pT4	—	—
Pre operative CEA	>200 ng/mL	—	—	≥200 ng/mL	>200 ng/mL
Pre operative Ca19.9	—	—	—	—	>200 ng/mL
*RAS/BRAF* status	—	—	—	—	Mutation
*mTBS*	—	—	—	—	5–12 (1 pts), ≥12 (2 pts)
Age	—	>60	—	—	—
		Risk Groups			
L	0–2	0–2	0–1	0	0–1
I	—	3–4	2–3	1	2–3
H	3–5	5–6	≥4	≥2	4–6

DFI, disease-free interval; LM, liver metastasis; CEA, Carcino-embryonic antigen; CA19.9, Carbohydrate Antigen 19-9; *RAS*, Rat Sarcoma gene; *BRAF*, B-Raf proto-oncogene; mTBS, modified Tumor Mutational Burden; L, low; I, intermediate; H, high.

In the last years, the survival of patients with CRLM has significantly improved, especially for “oligometastatic” patients. This term was coined in 1995 by Hellmann, defining patients with a limited number of metastatic deposits, and whose disease does not seem to progress to a widespread distribution of cancer [21]. For these patients, the combination of modern systemic therapies with loco-regional approaches, including advanced liver surgery and local ablative procedures, may be used with a curative intent [22].

Liver resection can change the natural history of this disease and is associated with prolonged survival also in patients with recurrence after resection [23]. Systemic chemotherapy, on the other hand, remains the only alternative in patients with unresectable CRLM, although prognosis is very poor. The 5 years overall survival (OS) rate of patients with CRLM is about 11% with palliative chemotherapy alone, while it reaches 50%–60% in those undergoing both systemic and local treatments [16].

A topic still debated in patients with S-CRLM and CRC is the optimal timing of surgery [24]. A *traditional approach* includes the resection of the primary tumor (especially in emergency presentation) followed by chemotherapy and, after 3–6 months of systemic treatment, completion with CRLMs resection. For selected patients, a *simultaneous resection* of CRC and CRLMs can be considered, despite a high risk of post-operative complications. Patients with asymptomatic CRC and initially unresectable or borderline resectable CRLMs, instead, are potential candidate for a *chemotherapy-first (or liver-first) approach,* including preoperative chemotherapy, followed by CRLM resection, adjuvant chemotherapy and resection of the primary tumor. Another scenario, to be preferentially offered to patients with asymptomatic primary tumors and initially resectable CLM, is the “*true*” *liver-first approach,* comprising upfront CRLM resection, followed by adjuvant chemotherapy, CRC resection and adjuvant chemotherapy. Since all of these are valid strategies, treatment plans must be personalized for each patient in multidisciplinary team meetings.

Although recent advances in molecular biology and the optimization of therapies in mCRC have progressively improved the survival of patients with CRLM, several aspects regarding the optimal therapeutic sequence as well as the use of targeted therapies remain to be clarified. Here, we review the current landscape of CRLM multimodal treatments with a particular focus on both loco-regional and systemic strategies as well as on the current limitations of the literature in this field.

## Locoregional Treatments of CRLMs

### Surgery

Radical resection of CRLM is widely accepted as treatment of choice for patients with resectable disease. Unluckily, approximately 10%–20% of CRLM are suitable d’amblée for this option [25]. The aim of surgery is to achieve a complete resection of both primary and metastatic sites with maximum sparing of the hepatic parenchyma [26]. Metastasis resection is defined R0 or “tumor-free” when complete removal of tumor is achieved with negative histological margins (>1 mm); R1 resection includes surgical margins ≤1 mm, while R2 resection refers to macroscopically incomplete resection [9].

The definition of LM resectability changed over time depending on clinical and radiological parameters, such as number, size and site of metastases, as well as node, vascular involvement and patient’s performance status [3]. In this context, the most important factor to define a patient with CRLM as resectable implies the maintenance of an adequate residual liver function after resection, considered as the preservation of at least two contiguous liver segments with sufficient blood flow, biliary drainage, and >20% residual liver of the total volume [27]. A residual volume liver ≥30% is required for patients who received chemotherapy, while ≥40% with liver fibrosis or cirrhosis [28]. The European Group for the Treatment of Colorectal Metastases (ECMTG) proposes that resection should be considered for patients with more than 30% post-surgery liver volume, and absence of either celiac lymph node involvement, unresectable extrahepatic disease, invasion of the hepatic hilum or the inferior vena cava, as well as the simultaneous involvement of all hepatic veins [3].

Both local recurrence rate (LRR) and prognosis of patients undergoing CRLM resection are affected by quality of surgery. The margins’ status plays the primary role in this context, R0 resections having the lowest risk of recurrence. A margin depth >1 mm is associated with a better prognosis than a submillimeter margin, while a margin >1 cm achieves the best outcome [29]. A free surgical margin, therefore, should be the goal of each CRML surgical treatment. The R1 resection, which occurs in nearly 10%–30% of liver resections [30], is associated with an increased LLR (9%–55% vs. 3%–8% with R0) [31–33]. When the CRLM is in contact with intrahepatic vessels, the risk of local recurrence depends by the type of vascular involvement, thus conditioning the surgical approach. The preservation of the hepatic veins, for example, is acceptable since it is associated with a low LRR, while the rescue of the Glissonean pedicle increases the risk of recurrence and should be avoided [34]. To this regard, a new distinction has been recently introduced between “parenchymal R1” (R1par)—or margin width <1 mm from CRLM—from “vascular detachment R1” (R1vasc), which describes a metastasis detached from first two orders of the Glissonean pedicles or from the hepatic pedicle veins in their last 4 cm before the confluence in the inferior vena cava. In terms of short and long oncological perspectives, the R1vasc is equivalent to R0 in obtaining local disease control and prolonged survival, while R1par is associated with a high risk of local recurrence and poor survival [35]. Several factors, including vascular proximity, multi-nodularity, or insufficient residual liver volume, significantly increase the risk of achieving a R1 resection.

Tumor biological factors also play a role in the risk of local recurrence. Although RAS mutations are associated with a more aggressive tumor biology [36], little is known about the association between RAS status, surgical margins and local recurrence in patients undergoing hepatectomy for CRLM. Unlike what happens in mutated KRAS (*mKRAS*), the margin status acquires a clear prognostic relevance in KRAS wild type (*wtKRAS*) CRLM [37, 38]. About long-term outcomes, tumor biology seems to influence the survival of patients with CRLM more than surgical margins [39]. Indeed, Margonis et al [40] found no differences in OS between R0 and R1 resections in mKRAS tumors, while OS following R0 resection was better than R1 in patients with *wtKRAS* tumors. However, both OS and hepatic-free survival (HFS) depends on the type of margin compensation regardless of KRAS status, although the differences between R0, R1vasc, and R1par are minor in *mKRAS* [30]. If confirmed, these data could strengthen vessel-sparing surgery in *wtKRAS* CRLM, while this policy should be adopted with caution in *mKRAS* patients.

### Local Ablation Techniques

Other locoregional liver treatments, including a number of interventional radiology ablative procedures, are now considered alternatives to surgery, or auxiliary treatment strategies in the multidisciplinary management of CRC metastases. The most used procedure includes the percutaneous thermal ablation [i.e., radiofrequency ablation (RFA) or microwave ablation (MWA)] and the chemo- or radio-embolization [41]. The RFA and MWA are widely accepted techniques for eliminating small CRLM, exploiting electromagnetic waves with different lengths and frequencies that cause cell necrosis. Image-guided percutaneous ablative therapies are indicated in patients with oligo-metastatic disease (≤4 CRLM), small dimension of LM (≤3 cm) or unsuitable for surgery (including patient’s refusal). The Amsterdam Colorectal Liver Met Registry (AmCORE) study analyzed safety, efficacy and survival outcomes after thermal ablation compared to partial hepatectomy for recurrent CRLM. It demonstrated that recurrent thermal ablations were not significantly different from recurrent partial hepatectomy in terms of survival. By contrast, there is a reduction in post procedural morbidity and mortality, length of hospitalization and costs, without compromising oncological outcomes [16]. The ongoing randomized phase III COLLISION trial (NCT030881590) should provide definitive answers regarding the non-inferiority of thermal ablation compared to liver resection in patients with at least one resectable and ablatable CRLM (≤3 cm) and no extrahepatic disease [42].

Radiofrequency ablation is the most commonly used locoregional procedure. It is a simple, repeatable, standardized, and low-risk procedure causing damage to cancer cells through frictional heating induced by high-frequency alternating current (375–500 kHz) in monopolar or bipolar radiofrequency systems [41]. Exposure of cancer cells to a temperature of approximately 50°C for 4–6 min induces cytotoxicity, while at 60°C–100°C cell proteins coagulate irreversibly causing coagulative necrosis. Temperatures above 100°C are rarely used since water evaporation and consequent drying result in electrical impedance limiting thermal transmission [43]. The efficacy of RFA is limited in patients with multiple CRLM and in those metastases close to large blood vessels that reduce the heat damage and attenuates cell death [44]. An excess margin of at least 5 mm is recommended to evenly surrounding the tumor and achieve good local tumor control [45].

More recently, MWA gained acceptance as a favorable and sometimes preferred alternative to RFA. This system uses microwave frequencies between 900 and 2,450 MHz to generate heat that causes cell death through coagulative necrosis [46]. The MWA has several advantages over RFA, such as higher intra-tumoral temperatures, faster heating on a larger volume of tissues and the possibility to use multiple applicators at the same time. Moreover, the MWA is not affected by either heat dissipation, high impedance, low thermal conductivity or low penetrability [47], thus the efficacy of MWA is not dampened in perivascular tumors and may be ideal for lesions close to vessels [48]. A randomized phase II clinical trial showed that MWA and RFA gain similar technical success and effectiveness in liver tumors between 1.5 cm and 4, in term of complications, median time to progression and OS [49].

Contraindications to the use of RFA include metastatic lesions >5 cm, ascites or perivascular tumors [50]. The ideal lesions for effective ablation are those <3 cm in maximum diameter, although some authors consider lesions up to 5 cm [41]. Despite the RFA of CRLMs adjacent to the gallbladder is considered relatively dangerous (risk for perforation and cholecystitis), it is feasible, effective and safe when performed with CEUS monitoring [51]. Finally, another myth to dispel is the contraindication to performing RFA in case of cardiac implantable electronic devices, such as cardiac pacemakers or implantable cardioverter defibrillators [52].

### Intra-Arterial Procedures

Other options for CRLM are intra-arterial therapies for patients with liver prevalent disease that are not candidate for surgery or other locoregional procedures. Hepatic trans-arterial chemoembolization (TACE) is a treatment that involves the infusion of drugs directly into the liver vasculature but is limited to treat metastases no more than 5–6 cm in diameter [53]. TACE aims to infuse chemotherapy drugs into small-caliber arteries of liver metastases, thus combining both ischemic and cytotoxic effect that lead to tumor cells’ death. Unlike other hyper-vascularized liver malignancies, such as hepatocellular carcinoma (HCC) and neuroendocrine tumor liver metastases, CRLMs receive a predominantly arterial vascular supply, thus making intra-arterial administration optimal for drug delivery [54]. In conventional TACE, lipiodol-emulsified chemotherapy agents (including irinotecan, oxaliplatin or doxorubicin) are injected with embolic particles, often polyvinyl alcohol or gelfoam, into the hepatic arteries supplying liver tumors while sparing the surrounding normal liver parenchyma [53]. In the last years, the introduction of TACE using drug-conjugated beads (DEB-TACE) significantly improved drug delivery into the tumor, while minimizing side effects [55].

Despite many studies have shown promising results with TACE of CRLMs, further understanding of its real-life clinical applications is still warranted. With this aim, the Cardiovascular and Interventional Radiological Society of Europe (CIRSE) initiated the CIRSE Registry for irinotecan-eluting LifePearl™ microspheres (LP-irinotecan) TACE. The primary objective of this wide prospective observational registry is to understand the real-life clinical application of LP-irinotecan TACE to ultimately determine at which stage of the disease the treatment is being conducted. Secondary objectives include treatment outcomes in terms of safety and efficacy. The first interim analysis on 50 patients revealed a prevalent use of LP-irinotecan TACE as salvage therapy (42%), while other applications included intensification treatment (20%), first-line treatment (16%), consolidation treatment (14%) or combined treatment with ablation with curative intent (8%). The analysis revealed an acceptable toxicity profile with most patients, except for those in salvage therapy, reporting a stable or improved health-related quality of life (HRQOL) than deterioration [56].

Other locoregional techniques, such as the trans-arterial radio-embolization (TARE), the hepatic arterial infusion (HAI) of chemotherapy as well as percutaneous hepatic perfusion (PHP, or chemosaturation) [55], are other specific treatments under investigation limited to a few centers. Particularly, TARE involves a single delivery of a radionuclide [yttrium (Y)-90, or holmium-166], connected to either resin/glass particles or bio-resorbable microspheres as delivery platform into the hepatic artery, which produce their therapeutic effect by irradiating the surrounding tissues [57]. Available data on TARE, however, are somewhat controversial. With the exception of a single small randomized study that supports the use of TARE for heavily pretreated patients with liver-limited metastases [58], other studies failed in the same intend. A large meta-analysis from three randomized studies [59] showed no benefit in OS when TARE was added to the first cycle of an investigator-determined “best systemic treatment.” Similar, a randomized phase III study of TARE failed to show a significant impact on survival, although a better “liver-specific PFS” was observed in patients with liver-limited or liver-predominant disease [59, 60]. In the second-line setting, a recent phase III trial (EPOCH) compared chemotherapy alone with chemotherapy plus Y-90 TARE in 428 patients with liver-dominant or liver-only disease, showing a significant improvement in PFS and ORR [61]. A subgroup analysis of this study suggests that patients with fewer than three lesions, resected primary tumor, lower tumor burden, left primary tumor location and *KRAS* mutation may benefit more from Y-90 TARE.

These latter options (Table 2) are currently considered potential approaches for patients who have previously failed systemic chemotherapy regimens and do not have other valid chances [53]. Their effective contribution to patient outcome, however, is still debated [62].

**TABLE 2:** Locoregional treatments of CRLMs.

Locoregional treatments			
	Type of treatment	Possible indications	Controindications
	Surgery	- Possibility of venous resection or reconstruction in case of vascular invasion - Favorable tumor location for surgery	- Portal lymphadenopathy
			- Lower resection margin of 1 mm free of tumor
			- Rate of future liver remnant <20% of total liver volume in normal livers, <30% in who received chemotherapy, <40% in liver fibrosis or cirrhosis
Local ablation techniques	RFA	- CRLM ≤ 4 - CRLM ≤ 3 cm in diameter	- CRLM >3 cm in diameter not candidates for surgical treatment
	MWA		- Localization close to hepatic vessels
			- Lesion margins < 5 mm (for local tumor control; it is better if the disease-free margins are greater than 10 mm)
Intra-arterial procedures	TACE	- CRLM no larger than 5–6 cm in diameter	- Several liver function alterations
	TARE		- Size and distribution of the tumor
			- High tumor burden
	HAI		- Comorbidities and poor performance status
			- Previous radiation therapy
	PHP		- Alteration of renal function
			- Complete thrombosis of the portal vein
Stereotactic body radiotherapy	SRBT	- CRLM ≤ 5	- >5 CRLM
		- CRLM ≤ 3 cm in diameter	- Failure to ensure the safety of liver function and adjacent organs e/o structures
		- PS ECOG ≤ 2	- PS ECOG > 2
		- Expected survival >3 months	- chemotherapy within 2 weeks of SBRT
		- < 700 cc of involved liver	
		- Potentially curable extrahepatic disease	

CRLMs, colorectal liver metastases; HAI, hepatic arterial infusion; MWA, microwave ablation; PHP, percutaneous hepatic perfusion; RFA, radiofrequency ablation; SBRT, stereotactic body radiotherapy; TACE, Transcatheter arterial chemoembolization; TARE, trans-arterial radio-embolization.

Globally, contraindications to intra-arterial procedures may include severe liver function alterations, presence of uncontrolled extrahepatic disease, complete thrombosis or involvement of the portal vein, and previous radiotherapy to the liver for TARE [63].

### Stereotactic Body Radiotherapy (SBRT)

Finally, in the context of oligometastatic disease, SBRT is presented as an alternative to other local therapies, to improve long-term disease control or possibly cure it. Several studies have demonstrated positive results in terms of safety, local control, OS and quality of life about this treatment [64–66] Favorable outcomes were related to appropriate patient selection and reasonable dose (Biologically effective dose ≥100 Gy_10_) [67] of radiotherapy (RT) administered to the targeted lesions. Advances in RT technology, diagnostics, and RT planning have increased treatment safety [68]. Different strategies have been developed with SBRT in order to solve issues relative to liver respiratory movements, including the use of abdominal compression, respiratory gating, four-dimensional computed tomography (4DCT) for the simulation’s procedures [69], implantation of fiducial markers for tumor tracking, and breath-hold methods [70]. In order to obtain a better definition of the target volumes, the simulation CT images are fused with magnetic resonance imaging (MRI) [71]. Compared to traditional normofractionated RT, SBRT allows for the precise delivery of possibly high ablative doses to liver metastases, sparing the uninvolved liver and surrounding critical structures as much as possible, to reduce the risk of RT-induced liver disease [72]. It is generally performed in 1–5 fractions [73], reducing overall treatment time to prevent treatment delays/interruptions of systemic therapy and to improve treatment response [68]. Based on the available data, hepatic SBRT should be evaluated for patients with oligometastatic, unresectable (for technical or medical reasons) CRLM, after failure of other local therapies and in combination with surgical resection. Patients should have an ECOG performance status of ≤2, expected survival >3 months, >700 cc of uninvolved liver, ≤5 liver metastases, potentially curable extrahepatic disease, adequate liver function (no cirrhosis Child C), and dimensions of the tumoral lesion < 6 cm [74]. Finally, the rate of local control in patients with *KRAS* and/or *TP53* mutation is relatively low (up to 20%) and this should be taken into consideration when choosing this approach [68].

The principal organ at risk of side effects in hepatic SBRT is liver itself. One of the potential hepatic toxicities of SBRT is radiation-induced liver disease (RILD), characterized by ascites, hepatomegaly, and elevated alkaline phosphatase within 4 weeks to 3 months after treatment [75]. Other late toxicities of hepatic SBRT include gastrointestinal (intestinal or duodenal-jejunal) bleeding/ulceration/perforation, soft-tissue (skin fibrosis) and bone (rib’s fractures) complications [76]. The adequate respect of dose constraints [77] and the systems to control the organ motion permit to reduce these possible complications.

New perspectives of SBRT for CRLM include the use of proton therapy to improve the sparing of normal tissues [78] and the MRI-guided linear accelerators for a better visualization of soft tissues and dynamic modification of treatment volumes based on daily anatomy changes and tumor response (adaptive RT) [79, 80].

Another interesting scenario is the synergy between SBRT and immunotherapy. In this context, SBRT has been shown to promote tumor antigens release and to initiate immune response, with the creation of a pro-inflammatory environment (activation of tumor-specific T cells, increasing immune modulator molecules), allowing immunotherapies to be more effective [81]. The combination of immune check-point inhibitors and SBRT has been extensively investigated in preclinical and early phase studies [82], but the understanding of the optimal dose and fractionation of RT to prime the immune system against metastatic colorectal cancer cells is still unknown; prospective trials are ongoing to try to answer to these exciting questions.

## Systemic Treatments

### Perioperative Chemotherapy for Resectable CRLM

Although still debated, the association of systemic therapies and surgery in initially resectable CRLM is mostly considered a standard of care [83]. Possible strategies include chemotherapy given prior to surgery, or as adjuvant treatment. A potential benefit of chemotherapy administered prior to surgery may be the possibility to “test” the aggressiveness of the tumor and avoid unnecessary surgery in patients with very poor prognosis. Responses to preoperative chemotherapy may also predict favorable prognosis, as reported by Chan et al that found a 5 years OS of 76% in patients with complete pathological response, compared to 45% with a partial response [84]. Another possible advantage of preoperative chemotherapy for resectable CRLM is to eradicate micro metastases prior to surgery [85]. By contrast, an adjuvant approach accelerates the start of surgery and reduce the risks of postoperative complications related to the deleterious effects of cytotoxic drugs. This is however counterbalanced by a not negligible increase in the risk of rapid progression during the immediate post-operative recovery phase [26].

A number of studies investigated the efficacy of a perioperative approach in patients with resectable CRLM. The EORTC intergroup trial 40983 (EPOC) randomized 364 patients with CRC and ≤4 LMs, comparing 6 cycles of perioperative FOLFOX (3 before and 3 after surgery) to surgery alone in initially resectable CRLM [86]. The trial showed an improvement in disease free survival (DFS) in the perioperative arm (20.9 versus 12.5 months), despite no significant advantage in OS [84, 87]. Compared to adjuvant chemotherapy in patients with resectable CRLM, however, perioperative FOLFOX did not improve either DFS or OS in a retrospective study [26].

Another crucial aspect of the perioperative systemic treatment concerns the use of monoclonal antibodies. The “New EPOC” phase III trial randomized 257 patients with *wtKRAS* tumors with resectable or borderline resectable LMs to receive chemotherapy (oxaliplatin plus fluoropyrimidine, or irinotecan plus fluorouracil) with or without the anti-EGFR monoclonal antibody cetuximab, before and after liver metastasis resection [85]. The trial was stopped due to the detrimental effect on PFS, which ultimately led to a shortening of OS [88]. Further *post hoc* analysis confirmed similar results also in the all-RAS (KRAS and NRAS) as well as the BRAF wild type population [89]. Interestingly, cetuximab was more harmful in subgroups associated with good prognostic characteristics (well or moderately differentiated primary tumors, fewer liver metastases, absence of N2 disease, and metachronous disease), that makes convincing the negative effect of anti-EGFR on this population [90]. Finally, the post-relapse survival was much worse in the group that received cetuximab, suggesting a development of aggressive disease phenotype at relapse or acquired resistance to cetuximab in post-relapse treatment approaches (failure to re-treat with anti-EGFR). Overall, these results make this treatment unsuitable in this setting [84].

The role of anti-angiogenics in this setting remains unclear. A small retrospective study showed the safety of chemotherapy regimens including bevacizumab (anti-VEGF) in the perioperative setting with 65.7% of objective responses and no negative impact on patient outcome [91]. The addition of bevacizumab to chemotherapy would not seem to increase liver complication rates after resection; moreover, patients with pathological complete response obtain longer OS, instead no difference in OS was observed between reponders and no-responders, without increase in term of morbidity or mortality related to liver resection [92].

### Adjuvant Chemotherapy After R0 Resection of CRLM

The aim of adjuvant chemotherapy is to reduce the risk of recurrence after surgery and increase cancer-specific survival. The use of oxaliplatin-based adjuvant chemotherapy is the standard of care for patients with high-risk stage II and stage III colon cancer with significant DFS and OS benefits [93]. However, the use of post-operative chemotherapy is controversial in patients with stage IV NED (No Evidence of Disease) following resection of primary tumor and CRLM. Even in R0 resection, in fact, the rate of recurrence within 2 years is about 75% [94].

Hepatectomy alone does not always provide a complete cure due to micro metastatic disease and adjuvant chemotherapy started within 3 months after liver resection warrants to reduce the odds of relapse. Possible chemo regimens are based on the association of fluorouracil and oxaliplatin (FOLFOX or CAPOX) for a duration of 6 months [95], while a fluoropyrimidine-based monotherapy (i.e., capecitabine) should be deserved for patients unfit for doublets, although a real benefit in OS has never been documented [96]. Different prospective trials (Table 3) compared intravenous adjuvant chemotherapy with observation alone after CRLM resection, although definitive results are lacking [97–99]. Three studies compared adjuvant fluoropyrimidine-based monotherapy in patients with stage IV NED to only observation after surgery. All these studies showed an improvement in mDFS (*Portier*: 24.4 vs. 17.6 months; *Mitry*: 27.9 vs. 18.8 months; *Hasegawa*: 17.4 vs. 8.4 months) but failed to confirm the efficacy on OS. Furthermore, the randomized JCOG0603 phase II/III trial compared hepatectomy alone to hepatectomy followed by 6 months of mFOLFOX6 in patients with liver-only metastatic CRC. This study confirmed a significant improvement in mDFS with mFOLFOX6, although the 5 years OS rate was superior in hepatectomy alone compared to hepatectomy followed by chemotherapy (83.1% vs. 71.2%, respectively) [100]. Mechanisms potentially explaining a similar detrimental effect of chemotherapy on OS are still ill-defined, but may be correlated with both chemotherapy-induced liver injury, as well as the selection of aggressive resistant tumor cell clones.

**TABLE 3:** Trials exploring adjuvant chemotherapy after CRLM resection.

Author	Patients (n)	Arms	mDFS (months)	5-OS (%)
Portier [97]	173	Chemotherapy (monotherapy: 5FU + LV) vs. observation	24.4 vs. 17.6	51.1 vs. 41.1
Mitry [98]	278	Chemotherapy (monotherapy: 5FU + LV) vs. observation	27.9 vs. 18.8	52.8 vs. 39.6
Hasegawa [99]	180	Chemotherapy (monotherapy: 5FU + LV) vs. observation	17.4 vs. 8.4	66.1 vs. 66.8
Kanemitsu [100]	300	Chemotherapy (mFOLFOX6) vs. observation	n.a.	71.2 vs. 83.1

5FU, 5fluorouracil; mDFS, median disease-free survival; LV, leucovorin; OS, overall survival.

Despite this, it remains unclear whether adjuvant chemotherapy improves OS in resected CRLM; it is generally accepted that patients with low-risk features (i.e., metachronous disease, limited number of metastases, R0) may be treated with surgery alone, while post-operative chemotherapy is generally recommended for high-risk patients (especially synchronous metastases, R1) and particularly those who did not receive adjuvant treatment for primary tumor resection.

### Conversion Chemotherapy for Primary Unresectable CRLM

The survival of patients with never resectable liver metastases is poor, with median OS after diagnosis of about 17 months, and 5 years OS rates <5% [101]. In the presence of primary unresectable CRLM, upfront chemotherapy may be considered to downstaging the metastatic burden and achieving resectability (conversion chemotherapy). In a wide study by Adam et al enrolling 1,104 patients with primary unresectable CRLM, conversion chemotherapy reverted to resectability about 12% of patients, with a 5 years survival rate of 33% [102]. Similar results are observed in patients undergoing upfront surgery for CRLM [103].

When considering conversion chemotherapy, many factors must be evaluated to attempt the best response to chemotherapy for a successful surgery, including mutational status, primary tumor sidedness and Tumor Burden Score (TBS). The TBS is based on radiographic features of CRLMs, involving tumor size and number of liver metastasis [104, 105]. Of note, the “low-TBS” has a 3-fold higher conversion rate than the “high- TBS”, which tends to have both worse objective response and conversion outcomes [106]. Different chemotherapy regimens can be used to convert the resectable state (Table 4), including doublet or triplet combinations (oxaliplatin- and/or irinotecan-based regimen) with or without targeted therapy, although the best regimen has not yet been defined. The phase III TRIBE [107] and phase II Olivia trials [108] showed a high resection rate in patients with liver-limited disease treated with a triplet regimen (FOLFOXIRI) ± bevacizumab, at the cost of increased toxicity. A pooled analysis by Tomasello et al including 11 studies (877 patients) with FOLFOXIRI-bevacizumab revealed a surgical conversion rate of 39% with 28.1% of R0 resections [109]. Similarly, FOLFOX6-bevacizumab was associated with a 23.1% rate of surgical conversion, including 15.4% of R0. The TRICC0808 trial revealed long survivals (median 36.8 months) in patients treated with hepatectomy after mFOLFOX6 and Bevacizumab, although most of the patients developed recurrence [110]. The CELIM and PLANET phase II trials also demonstrated favorable long-term survivals in patients with initially unresectable *wtKRAS* CRLM who responded to anti-EGFR antibody-based conversion therapies and underwent liver resection [112]. Although no definitive data about which regimen should be preferred, there is sufficient evidence that patients with left-sided primary tumors benefit more than right-sided from the use of EGFR-antibodies with doublet chemotherapy in terms of response rate (RR), overall survival (OS) and progression free survival (PFS) [113]. The phase III PARADIGM trial was the first prospective study confirming the superiority of panitumumab (anti-EGFR monoclonal antibody) plus mFOLFOX6 vs. bevacizumab plus mFOLFOX6 as first-line treatment in patients with wtRAS mCRC and left-sided primary tumor in terms of OS (mOS 37.9 vs. 34.3 months) [114]. In the recent final analysis of this trial, the Authors showed that panitumumab added to first line chemotherapy in left-side tumors allowed a curative resection rate of 18.3% compared to 11.6% with bevacizumab plus chemotherapy [111]. Therefore, both anti-EGFR regiments and triplets should be considered whenever the aim is to convert liver metastasis to resectability. Recently, first-line immunotherapy with pembrolizumab (anti-PD1) in patients with microsatellite instability-high (MSI-H) or deficient mismatch repair (dMMR) mCRC obtained unprecedent PFS and OS as compared to chemotherapy in the KEYNOTE-177 phase III trial [115]. Immunotherapy in locally advanced MSI-H CRC also overwhelmed with impressive rates of complete response in the neoadjuvant setting, thus potentially changing the treatment strategy in these patients [116, 117]. In this context, immunotherapy is characterized by durable and deep responses [118], thus the role of liver metastasectomy in patients with *MSI-H/dMMR* mCRC is to be reconsidered since a non-operative approach and a surveillance-based management may be more appropriate [119].

**TABLE 4:** Trials exploring conversion chemotherapy for unresectable CRLM.

Author	Patients	Arms	Conversion rate (%)	R0 surgery (%)
Cremolini [107]	508	FOLFOXIRI + Bevacizumab vs. FOLFIRI	n.a.	16.4 vs. 11.8
Gruenberg [108]	80	FOLFOXIRI + Bevacizumab vs. mFOLFOX6 + Bevacizumab	61 vs. 49	49 vs. 23
Tomasello [109]	877	FOLFOXIRI + Bevacizumab	39.1	28.1
Yasuno [110]	45	mFOLFOX6 + Bevacizumab	23.1	44.4
Watanabe [111]	823	mFOLFOX6 + Bevacizumab vs. mFOLFOX6 + Panitutmumab	68.6 vs. 80.2 (left-side) 67.3 vs. 74.9 (overall)	11.6 vs. 18.3 (left-side) 10.9 vs. 16.5 (overall)

Once the conversion therapy achieves its aim and metastasis resection becomes feasible, chemotherapy should be promptly discontinued to avoid unnecessary liver toxicity [120] and preserve liver residual function. The hepatic sinusoidal obstruction syndrome (SOS), in example, is an obliterative venulitis of the terminal hepatic veins following oxaliplatin administration which, in severe cases, has a high risk of mortality [121]. Also, steatosis and steatohepatitis can occur with both oxaliplatin and irinotecan use with a 10-fold increase of post-operative morbidity [122]. The addition of targeted therapies (anti-VEGF or anti-EGFR) to conventional chemotherapy does not increase the postoperative morbidity and mortality rates after hepatectomy [123], while a protective effect of bevacizumab against SOS induced by oxaliplatin-based chemotherapy has also been described [124]. Noteworthy, chemotherapy-induced liver injuries condition a poorer short-term prognosis [125] and is responsible for 20%–25% increase of post-surgical complications as compared to patients receiving surgery alone [126, 127]. The safety of these patients mainly depends on a careful preoperative evaluation of liver volumes and limited use of cytotoxic agents followed by 5-week break before surgery [124, 128]. Not the least, continuing chemotherapy beyond the attainment of a resectable state may induce the phenomenon of the “vanishing liver metastases” for which they become undetectable for subsequent surgery [129].

## Conclusion and Future Perspectives

**FIGURE 1:**
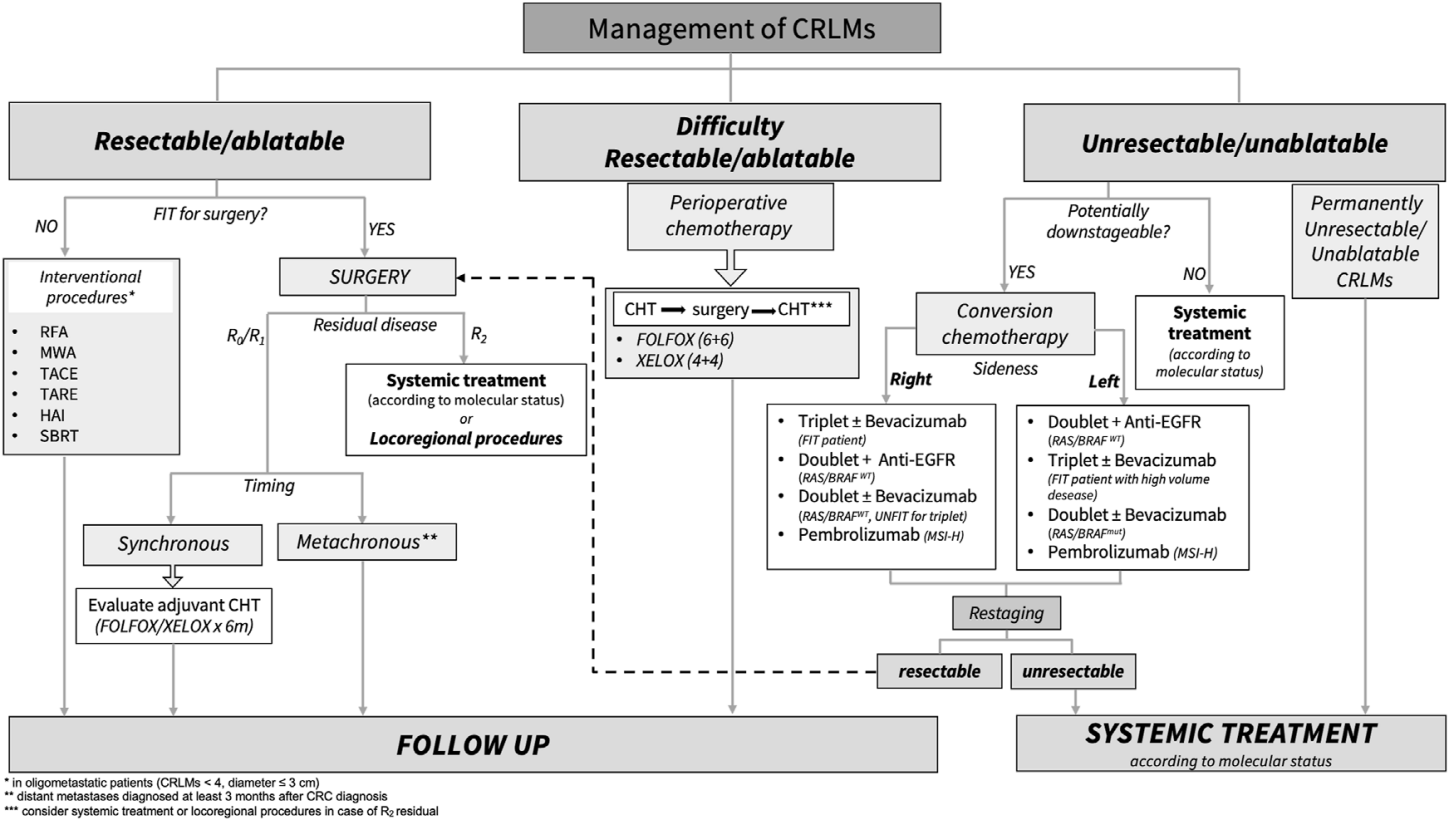
Standard treatment algorithm for patients with colorectal liver metastasis. CRLMs, colorectal liver metastasis; RFA, radiofrequency ablation; MWA, microwave ablation; TACE, hepatic trans-arterial chemoembolization; TARE, trans-arterial radio-embolization; HAI, hepatic arterial infusion; SBRT, stereotactic body radiation; Anti-EGFR, Panitumumab/Cetuximab; MSI-H, microsatellite instability high; WT, wild type; Mut, mutation; CHT, chemotherapy.

The treatment of choice for resectable CRLM is surgery with the aim of R0 resection. In selected cases, patients with oligometastatic disease also advantage from loco-regional approaches (es. RFA, TACE, SBRT, etc.), achieving a modest probability of oncological radicality. Systemic chemotherapy is safe both peri-operatively, for primarily resectable metastases, and pre-operatively (conversion therapy) to achieve resectability of primary unresectable CRLM. While the only accepted regimen for peri-operative approach is the association of fluoropyrimidines and oxaliplatin, the regimen for a conversion therapy should be based on the intention of obtaining the greatest probability of response, therefore defined in relation to primary tumor sidedness, molecular features, and clinical characteristics. After CRLM resection, the role of adjuvant chemotherapy is controversial. The poor benefit in terms of survival needs further improvements in the selection of patient that are amenable to chemotherapy. Figure 1 summarizes the therapeutic decision algorithm in the management of patients with CRLM.

Further knowledge and innovative technologies are needed to customize treatment decisions in CRLM. In this context, the application of circulating tumor DNA (ct-DNA) has the potential to be informative as seen for early-stage CRC (stage II or III) to detect the “Minimal Residual Disease” (MRD) [130, 131]. The ct-DNA, in example, may be useful in resected CRLM patients to select those with high risk of recurrence to candidate for adjuvant therapy [132–134]. Moreover, ct-DNA could also be used in patients with CRLM undergoing perioperative or conversion therapy to evaluate the appropriate timing of liver surgery, as proposed in a recent prospective study [135].

Comprehensively, the best results, in terms of survival and quality of life, for patients with CRLM are certainly obtained by appropriate multimodal approach and multidisciplinary management. It is conceivable that the concomitant use of systemic therapies and loco-regional procedures, when adequate and managed by a team of experts, should be considered whenever is possible.
